# Aging‐related morphological changes in the obturator internus and levator ani

**DOI:** 10.1111/joa.70256

**Published:** 2026-10-19

**Authors:** Satoru Muro, Junichi Tsuchiya, Takuya Ibara, Akimoto Nimura, Keiichi Akita

**Affiliations:** ^1^ Department of Clinical Anatomy, Graduate School of Medical and Dental Sciences Institute of Science Tokyo Tokyo Japan; ^2^ Department of Diagnostic Radiology and Nuclear Medicine Institute of Science Tokyo Tokyo Japan; ^3^ Biomedical Engineering Laboratory, Department of Functional Joint Anatomy, Institute of Industry Incubation Institute of Science Tokyo Tokyo Japan

**Keywords:** aging, cross‐sectional studies, magnetic resonance imaging, pelvic floor, pelvic floor disorders, skeletal muscle

## Abstract

Pelvic floor dysfunction is a common condition in adult women. In addition to the levator ani, the obturator internus, a lateral pelvic wall muscle, has been suggested to be anatomically and mechanically associated with pelvic floor morphology. Here, we quantitatively characterized age‐related morphological changes in the obturator internus and levator ani using geometric morphometric analysis. We hypothesized a significant association between principal components accounting for major shape variation and age. This cross‐sectional study included 27 adult women aged 25–83 years with gynecological conditions but without pelvic floor disorders based on clinical assessment and imaging findings. Standardized pelvic magnetic resonance imaging scans were obtained between April 2024 and July 2024 at a single tertiary care center. Elliptic Fourier analysis was applied to the segmented obturator internus contours; a landmark‐based method was used to evaluate the combined shape of the obturator internus and levator ani. Principal component analysis and linear regression were performed to assess associations with age. Procrustes analysis of variance was used to evaluate the overall shape–age relationships. Elliptic Fourier analysis revealed that increasing age was significantly associated with relative narrowing of the superior portion, an inferior morphological shift of the medial surface groove, and increased prominence of the medial eminence of the obturator internus. A landmark‐based analysis revealed similar age‐related shape changes, including inferiorly directed shape changes of the groove, verticalization of the levator ani, and widening of the levator hiatus. Procrustes analysis revealed a significant relationship between age and overall shape variation. Aging was associated with coordinated age‐related morphological remodeling of the obturator internus and levator ani.

## Graphical abstract

A cross‐sectional MRI‐based morphometric analysis of 27 women aged 25–83 years revealed age‐associated morphological differences in the obturator internus and levator ani. Older age was associated with relative superior narrowing of the obturator internus, inferior displacement of its groove and medial eminence, a more vertical orientation of the levator ani, and widening of the levator hiatus. These findings suggest coordinated age‐associated morphological remodeling of the obturator internus and levator ani.

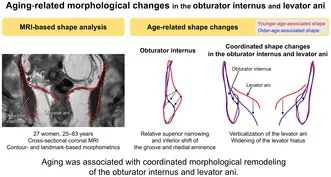

## 1 INTRODUCTION

Pelvic floor dysfunction is associated with aging, childbirth, and postoperative changes following pelvic surgery. In women, the prevention and treatment of pelvic organ prolapse (POP) and urinary incontinence remains an urgent issue from both clinical and societal perspectives (Milsom & Gyhagen, 2019; Richter et al., 2010; Wu et al., 2014). Traditionally, research on the pathophysiology of pelvic floor dysfunction has focused on morphological changes in the levator ani and puborectalis muscles. More recently, attention has focused on the potential involvement of adjacent hip joint muscles, particularly the obturator internus (Okumura et al., 2017; Tuttle et al., 2016). The obturator internus is anatomically contiguous with the levator ani through a broad fascial interface (Muro et al., 2023). A comprehensive evaluation of age‐related pelvic floor morphology may benefit from the inclusion of both the levator ani and obturator internus.

Previous studies on age‐related morphological changes of the pelvic floor musculature, particularly the levator ani, have primarily utilized linear or area‐based metrics, such as muscle volume, cross‐sectional area, and positional measurements. Age‐related changes in the levator ani and obturator internus reduce the physiological cross‐sectional area (PCSA) and muscle volume (Cook et al., 2017; Morris et al., 2012; Wente & Dolan, 2018). The obturator internus is not a homogeneous, disc‐shaped muscle, but rather composed of two regions—superior and inferior—with distinct fiber orientations, forming a characteristic medial “groove” structure on its medial surface (Muro, Tsuchiya, et al., 2026). This groove approximately corresponds to the anatomical origin of the levator ani and represents a distinct morphological feature of the medial surface of the obturator internus. Although the vertical position of the groove demonstrated a weak negative correlation with age, this association was not significant (Muro, Tsuchiya, et al., 2026). This lack of statistical significance may, in part, reflect the limitations of single linear parameters, such as the superior–inferior position of the groove, in capturing the complex structural variation underlying age‐related morphological changes.

To overcome the methodological limitations that have led to a poor understanding of age‐related morphological changes in the obturator internus and levator ani, we aimed to quantitatively analyze these changes using geometric morphometrics (GM). This multivariate analytical technique captures shape configurations in their entirety and has recently gained prominence in human anatomical research due to its ability to quantify subtle morphological variations (Muro, Kim, et al., 2024). We hypothesized that the principal components (PCs) that contribute the most to overall shape variation would significantly correlate with age, reflecting the predominant patterns of age‐related morphological changes in the obturator internus and levator ani. This study provides novel insights into age‐related morphological changes of the levator ani and obturator internus and offers a morphological framework that may inform future research on the assessment and management of pelvic floor dysfunction.

## 2 MATERIALS AND METHODS

### 2.1 Participants

In this retrospective cross‐sectional study, we utilized pelvic MRI images acquired between April 2024 and July 2024 at our institution. The participants were women with gynecological conditions, aged 25–83 years, with a median age of 54 years (interquartile range, 47–66 years). The gynecological conditions included benign gynecological diseases without clinical or radiological evidence of pelvic floor dysfunction. The absence of pelvic floor dysfunction was confirmed through a combined review of medical records and magnetic resonance imaging (MRI) findings. Patients with a history of pelvic organ prolapse, stress urinary incontinence, rectocele, prior pelvic floor surgery, or pelvic organ descent below the pubococcygeal line on sagittal MRI were excluded, and so were those with considerable motion artifacts, adhesions, or tumor invasion involving the obturator internus or levator ani muscles.

Cases with significant motion artifacts or adhesions that impeded reliable identification of muscle contours were excluded. Finally, the MRI data from 27 consecutive cases were analyzed. This dataset was identical to that used in a previous study (Muro, Tsuchiya, et al., 2026).

This study was conducted in accordance with the ethical standards of the Declaration of Helsinki and was approved by the Institutional Review Board (IRB) of the Institute of Science Tokyo (approval number: M2024‐091). The IRB waived the need for informed consent under an opt‐out policy. All data were anonymized. Information on parity, menopausal status, body mass index, hormonal therapy, physical activity level, and pelvic floor muscle strength was not consistently available and was therefore excluded from the analysis.

### 2.2 MRI acquisition and image standardization

Pelvic MRI was performed with patients in the supine position using coronal T2‐weighted sequences (echo time, 85–118 ms; repetition time, 3000–5500 ms). Imaging was conducted using 1.5‐T (MAGNETOM Sola; Siemens Healthineers, Erlangen, Germany) and 3.0‐T (Signa Pioneer; GE Medical Systems, Milwaukee, WI, USA) MRI scanners. The slice thickness was 4 mm, with an interslice gap of 0.4 mm. The field of view was set to 25 cm, and the matrix size ranged from 384 to 432 × 268 to 300. Despite differing field strengths, standardized T2‐weighted protocols ensured comparability across scans.

Standardized coronal sections of the pelvis were obtained using anatomical landmarks as previously described (Muro, Tsuchiya, et al., 2026). Specifically, the bilateral pubic tubercles and anterior superior iliac spines were used as reference points. Fiducial markers were placed at these four landmarks using 3D Slicer (version 5.6.2 r32448), and coronal planes passing through all four points were reconstructed using the Reformat tool (Registration > Specialized > Reformat module). This anatomically standardized plane was used to reduce between‐participant variation in section orientation and to facilitate consistent contour extraction and landmark placement; it does not, by itself, establish landmark‐placement reliability.

### 2.3 Elliptic Fourier analysis of the obturator internus

For shape analysis of the obturator internus alone, standardized MRI coronal sections on the right side were used (*n* = 27). Obturator internus contours were extracted using Seg&Ref, a semiautomatic segmentation tool developed by our group (Figure 1a) (Muro et al., 2025; Muro, Ibara, et al., 2026). Only the right obturator internus was analyzed to ensure statistical independence across participants. The extracted contours were subjected to shape analysis using elliptic Fourier descriptors (EFDs). We employed the SHAPE software (version 1.3; Hiroyoshi Iwata) for the analysis (Iwata & Ukai, 2002).

**FIGURE 1:**
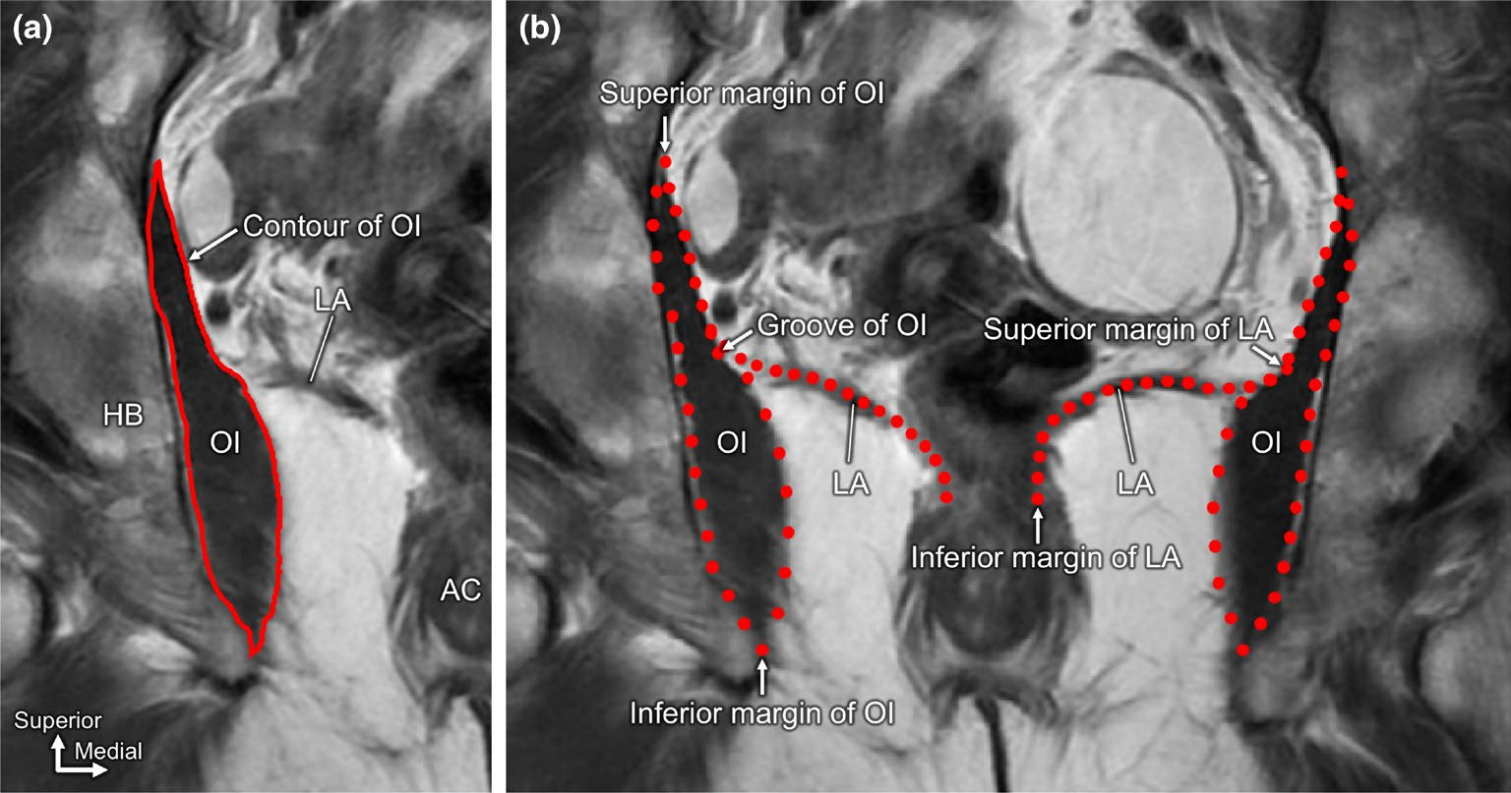
Methods of shape analysis applied to the obturator internus and levator ani muscles. (a) Semiautomatic segmentation of the obturator internus on standardized coronal magnetic resonance imaging (MRI) images using the Seg and Ref tool. The extracted contour was used for elliptic Fourier analysis. (b) Landmark configuration for geometric morphometric analysis of the obturator internus and levator ani muscles. A total of 98 landmarks were placed bilaterally according to predefined muscular landmarks. AC, anal canal; HB, hip bone; LA, levator ani; OI, obturator internus.

This software automatically performs the following steps: (1) contour extraction from image files, (2) computation of EFDs, (3) standardization of contour size and orientation, (4) PCA based on EFD coefficients, and (5) shape reconstruction corresponding to each PC. Principal component analysis was performed on the contours of the obturator internus extracted from MRI images using EFDs.

### 2.4 Landmark‐based morphometric analysis of the obturator internus and levator ani

Two‐dimensional coronal MRI representations of the bilateral obturator internus and levator ani muscles were used for their combined two‐dimensional morphological analysis. Landmark placement was performed on the coronal plane using tpsUtil and tpsDig2 (F. J. Rohlf, SUNY Stony Brook). Landmarks were defined according to the following anatomical criteria: (1) superior margin of the obturator internus; (2) inferior margin of the obturator internus; (3) characteristic “groove” on the medial surface of the obturator internus; (4) superior margin of the levator ani; and (5) inferior margin of the levator ani, defined as the interface between the levator ani and external anal sphincter on coronal MRI. The landmarks were evenly distributed between these structural features as follows: 15 points between the superior and inferior margins of the obturator internus, 7 between the superior margin and groove, 7 between the inferior margin and groove, and 15 between the superior and inferior margins of the levator ani, resulting in 49 landmarks on each side and 98 landmarks bilaterally (Figure 1b). A single trained evaluator placed all landmarks using the predefined criteria and a fixed landmark order. These procedures were intended to reduce observer‐dependent variation; however, formal inter‐ and intra‐observer reliability testing was not performed, and landmark‐placement accuracy cannot therefore be quantified.

Shape analysis was conducted using the geomorph package (version 4.0.10) in R using RStudio. Generalized Procrustes analysis (GPA) was first applied to remove variations due to translation, scale, and rotation. Principal component analysis was performed on aligned landmark configurations.

### 2.5 Statistical analysis

All statistical analyses were performed in R using RStudio. Two complementary morphometric analyses were conducted. The first was an elliptic Fourier analysis (EFA) of the obturator internus alone, which quantitatively captured the contour shape of the obturator internus on the right side. The second was a landmark‐based GM analysis of the bilateral obturator internus and levator ani, evaluating the relative configuration of these muscles in the coronal plane. These two analyses were designed to capture different aspects of pelvic floor morphology: EFA focused on local contour deformation of the obturator internus, whereas the landmark‐based approach assessed the overall two‐dimensional configuration of the obturator internus and levator ani within the coronal plane.

For each analysis, PCA was applied to the shape data of the obturator internus and levator ani, and the resulting PC scores were used as quantitative indicators of morphological variation. To ensure interpretability and avoid overfitting, only PCs that together explained approximately 80% of the total shape variance were retained for subsequent analyses. Principal component score normality was assessed using the Shapiro–Wilk test, and results were confirmed using permutation‐based methods where appropriate. Accordingly, parametric linear regression models were applied. To evaluate age‐related shape changes, simple linear regression analyses were conducted with age as the explanatory (independent) variable and each PC score as the response (dependent) variable. Regression coefficients, p‐values, coefficients of determination (*R* ^2^), residual standard errors, and F‐statistics were calculated to assess statistical significance.

Procrustes analysis of variance was performed using the procD.lm function on the generalized Procrustes analysis‐aligned landmark coordinates to examine the association between age and overall shape variation; statistical significance was assessed using 1000 permutations. Cohen's *f* ^2^ for each regression model was calculated as *R* ^2^/(1 – *R* ^2^). For the two statistically significant simple regression models, post hoc power was estimated from the noncentral F distribution using one predictor, a sample size of 27, a two‐sided alpha level of 0.05, numerator degrees of freedom of 1, and denominator degrees of freedom of 25. Statistical significance was set at *p* < 0.05, and effect sizes and permutation‐based *p*‐values were reported where applicable.

### 2.6 Sample size justification

This study was designed as an exploratory geometric morphometric analysis and included all consecutive eligible cases within a fixed 4‐month acquisition window (April–July 2024). No a priori power calculation was performed. For the two statistically significant associations, the observed effect sizes of *f* ^2^ = 0.33 and *f* ^2^ = 0.52 corresponded to post hoc powers of 0.82 and 0.95, respectively. These estimates describe sensitivity for the observed effects and should not be interpreted as a substitute for prospective sample‐size determination.

## 3 RESULTS

### 3.1 Shape variation of the obturator internus: Elliptic Fourier analysis

The cumulative contribution of the first three principal components was 79.19%, accounting for most of the shape variation, with individual contribution rates of 53.24%, 15.69%, and 10.26% for the first, second, and third principal components, respectively (Figure 2). The first principal component primarily reflected proportional shape variation of the obturator internus, including relative narrowing of the superior portion and variation in the relative vertical positions of the medial groove and inferior medial eminence. In contrast, the second and third principal components captured variations in the contour of the medial surface, particularly the relative depth of the groove and prominence of the medial eminence.

**FIGURE 2:**
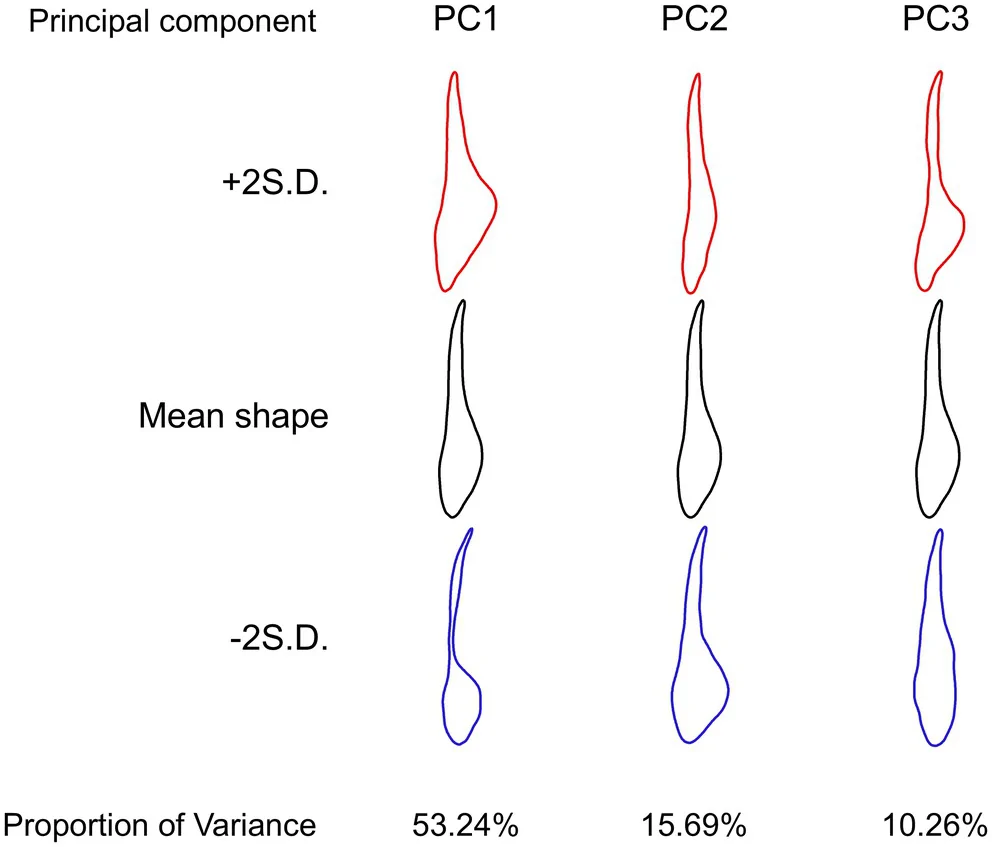
Principal component analysis (PCA) of the obturator internus based on elliptic Fourier descriptors. The cumulative contribution of the first three principal components accounts for 79.19% of the total shape variation. The first principal component captures proportional contour‐shape variation and vertical features, including relative narrowing of the superior portion and variation in groove position, whereas the second and third principal components reflect contour variation on the medial surface.

Regarding age‐related changes, PC1 showed a significant negative correlation with age (estimate = −0.00193, *p* = 0.008, *R* ^2^ = 0.25, *f* ^2^ = 0.33, post hoc power = 0.82) (Figure 3). In contrast, no significant association was found between age and PC2 or PC3 scores (PC2, *p* = 0.46, *R* ^2^ = 0.02; PC3, *p* = 0.068, *R* ^2^ = 0.13). The third principal component showed a non‐significant tendency toward a negative association with age.

**FIGURE 3:**
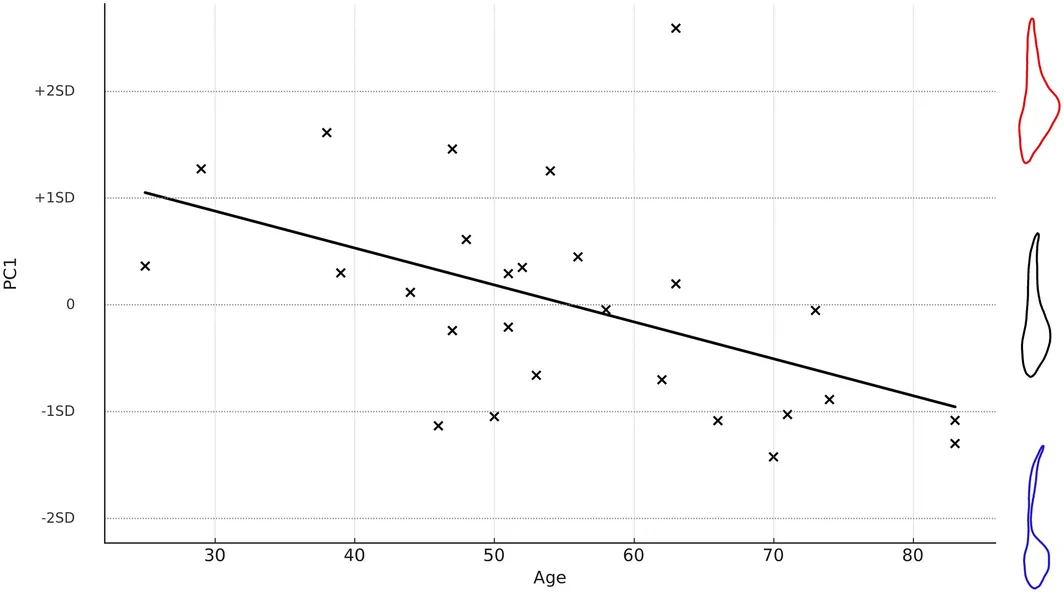
Association between PC1 score and age in the elliptic Fourier analysis. Linear regression revealed a significant negative correlation between age and the first principal component score (*p* = 0.008, *R* ^2^ = 0.25), indicating an age‐related downward shift in the medial groove and relative narrowing of the superior portion of the obturator. OI, obturator internus.

### 3.2 Morphological changes of the obturator internus and levator ani: Landmark‐based analysis

The cumulative contribution of the first two PCs was 62.8%, with PC1 accounting for 40.16% and PC2 accounting for 22.61%. The first principal component primarily reflected coordinated vertical shape variation of the medial groove and medial eminence of the obturator internus, inclination of the levator ani, relative vertical shape features of its inferior margin, and the relative medial distance between the bilateral levator ani. The second principal component was associated with the curvature of the medial eminence of the obturator internus, relative vertical shape features of the superior margin of the levator ani, and the relative width of the levator hiatus (Figure 4). Although PC3 contributed to the shape variation (14.35% of the variance), it is omitted from the graphical presentation for clarity.

**FIGURE 4:**
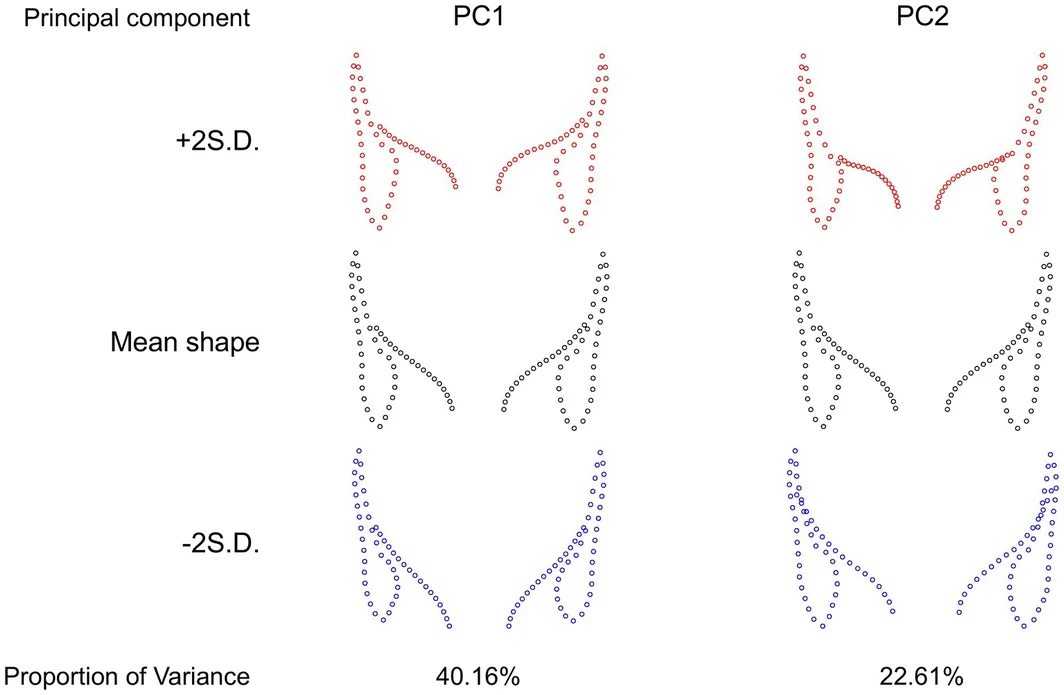
Principal component analysis (PCA) of the obturator internus and levator ani based on landmark‐based morphometrics. The first and second principal components cumulatively account for 62.8% of the shape variation. The first principal component reflects vertical displacement of the obturator internus groove and medial eminence, levator ani inclination, and levator hiatus width. The second principal component captures variation in the curvature and relative positions of the levator ani and obturator internus.

Linear regression analysis revealed a statistically significant negative association between PC1 scores and age (estimate = −0.00283, *p* = 0.001, *R* ^2^ = 0.34, *f* ^2^ = 0.52, post hoc power = 0.95; Figure 5). Furthermore, Procrustes ANOVA conducted using the GPA‐aligned landmark coordinates showed that age accounted for approximately 15.1% (*R* ^2^ = 0.15) of the total shape variation (*F* = 4.44, *p* = 0.003, *Z* = 2.82).

**FIGURE 5:**
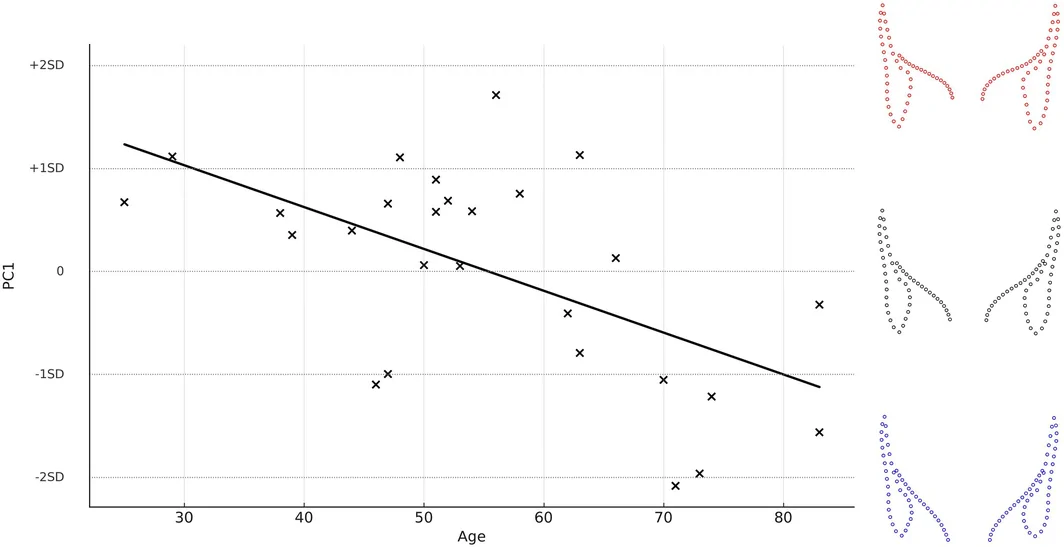
Association between PC1 score and age in the landmark‐based analysis. Linear regression analysis showed a significant negative correlation between age and the first principal component score (*p* = 0.001, *R* ^2^ = 0.343), suggesting coordinated age‐related structural differences in the obturator internus and levator ani, including vertical displacement of the obturator internus groove and medial eminence, downward displacement of the levator ani, and widening of the levator hiatus.

## 4 DISCUSSION

Elliptic Fourier analysis revealed that the coronal sectional shape of the obturator internus changed with age and was characterized by relative narrowing of the superior portion and an inferiorly directed shape variation of both the medial groove and the medial eminence (Figure 6a). Landmark‐based analysis corroborated these findings by demonstrating concordant inferiorly oriented shape variation in the groove and the eminence, along with concurrent structural changes, such as verticalization of the levator ani and widening of the levator hiatus (Figure 6b). To the best of our knowledge, this is the first study to apply geometric morphometric methods to quantitatively assess age‐associated shape configuration differences of the obturator internus and levator ani. Because the participants had no clinically evident pelvic floor dysfunction, these findings should be interpreted as age‐associated muscular remodeling rather than disease‐specific displacement.

**FIGURE 6:**
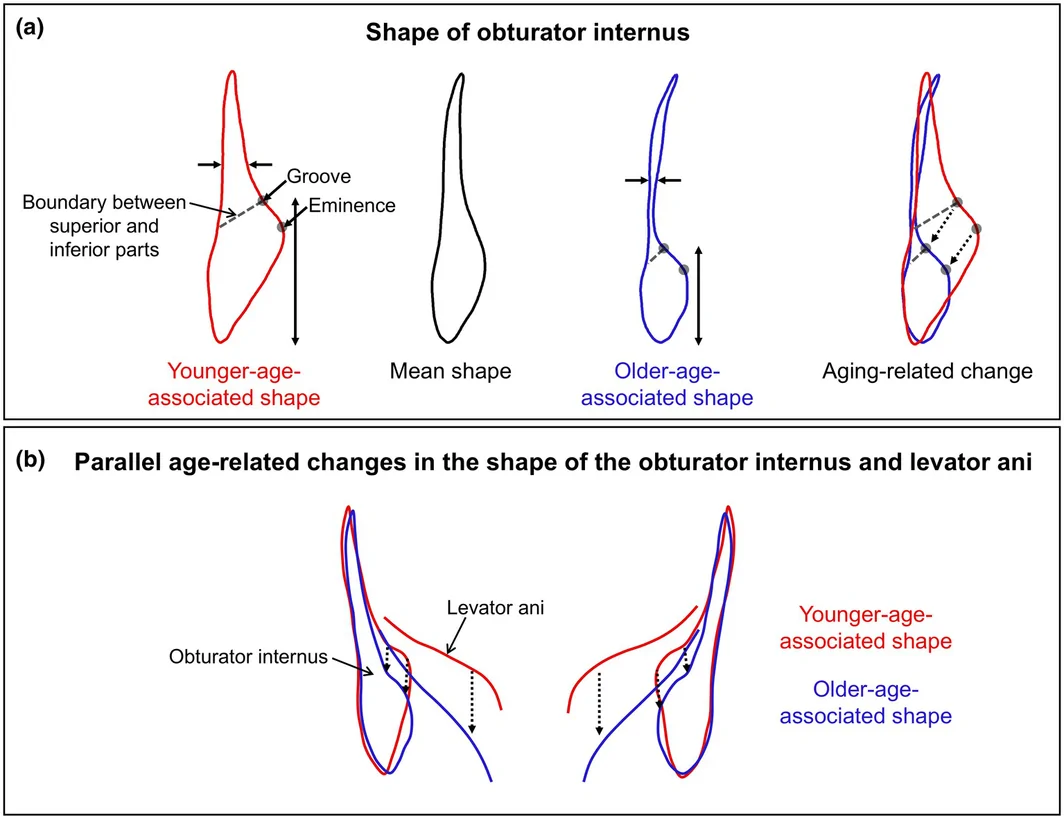
Representative shape changes associated with age. (a) Reconstructed shapes from elliptic Fourier analysis illustrate age‐related morphological trends in the obturator internus. With increasing age, the medial groove and inferior eminence of the obturator internus shift inferolaterally. The superior portion becomes relatively narrower, whereas the inferior portion shows a relative reduction in vertical extent. (b) Landmark‐based reconstructions show coordinated age‐related shape differences in the obturator internus and levator ani. The groove and eminence of the obturator internus descend inferiorly, accompanied by downward displacement of the superior and inferior margins of the levator ani. The levator ani shifts downward, becomes more vertically oriented, and the levator hiatus widens.

The levator ani is a morphologically and functionally heterogeneous muscle complex. Its anatomy has been described using pubovisceral (pubococcygeal), puborectal, and iliococcygeal components, although terminology varies among anatomical studies (Kearney et al., 2004; Muro, Moue, & Akita, 2024). Coordinated action of these components contributes to pelvic organ support and to narrowing and opening of the pelvic outlets during continence, urination, and defecation (Petros & Ulmsten, 1997). Because the present coronal MRI analysis treated the levator ani as a single muscle configuration, the observed verticalization and hiatus widening cannot be attributed to a particular subdivision.

Pelvic floor support has also been investigated from perspectives emphasizing ligamentous and connective tissue support (DeLancey et al., 2024; Petros & Ulmsten, 1993). Age‐ and childbirth‐related alterations in these supporting tissues may contribute to pelvic floor dysfunction independently of, or in combination with, muscular changes. However, ligamentous structures, obstetric history, clinical symptoms, and surgical outcomes were not evaluated in the present study. Accordingly, the observed age‐associated muscular morphology should not be interpreted as establishing a causal mechanism for pelvic organ prolapse or urinary, bowel, or pain symptoms, and the relationships among muscular, ligamentous, and connective tissue changes remain to be investigated.

Previous studies on age‐related changes in the pelvic floor muscles have predominantly focused on alterations in the position of the levator ani or inclination (Duarte Thibault et al., 2023; Okuda et al., 2025; Swenson et al., 2020). The present study provides a novel structural and quantitative assessment of age‐related shape alterations of the medial surface of the obturator internus, representing a significant advancement in understanding pelvic floor muscle aging and offering a new perspective and methodology for morphological analyses in this anatomical domain. Our finding of age‐related shape variation in the groove and medial eminence on the medial surface of the obturator internus parallels previously reported observations, suggesting a potential association between obturator internus morphology and pelvic floor‐related function (Muro et al., 2023). Prior research has extensively examined age‐related morphological changes in the pelvic floor musculature (Cheng et al., 2022; DeLancey et al., 2024). However, studies on obturator internus have largely focused on volumetric or cross‐sectional indices. These studies reported age‐related decreases in muscle thickness and PCSA (Cook et al., 2017; Morris et al., 2012; Tienza et al., 2015; Wente & Dolan, 2018) but did not address morphological shape changes. We previously identified a characteristic groove along the medial surface of the obturator internus (Muro, Tsuchiya, et al., 2026) and posited that such localized features require multivariate and geometric approaches for accurate characterization (Muro, Kim, et al., 2024). Therefore, we employed two GM methods—EFA and landmark‐based analysis—to demonstrate that age‐related shape changes in the obturator internus occur in parallel with the verticalization of the levator ani and widening of the levator hiatus. In particular, the inferior displacement of localized structures revealed age‐related morphological alterations that could not be captured by traditional linear indices.

In men undergoing prostatectomy, greater preoperative obturator internus thickness was significantly associated with earlier postoperative recovery of urinary continence (Hashimoto et al., 2025). In women with POP, the cross‐sectional area of the obturator internus is significantly smaller than that of controls, implying that obturator internus atrophy may be a risk factor for POP development (Sammarco et al., 2021). Additionally, patients with POP exhibit an increased anterior pelvic cross‐sectional area (Sammarco et al., 2019). Several reports have demonstrated improvements in urinary incontinence among women after undergoing total hip arthroplasty (THA) (Okumura et al., 2017; Tamaki et al., 2014). Posterior approaches to THA can damage the obturator internus, and muscle atrophy in this region has been associated with worsening urinary symptoms (Baba et al., 2014). These observations suggest that among the hip muscles, the obturator internus may be structurally relevant to pelvic floor morphology. Anatomically, this functional linkage is underscored by the fascial interface between the medial surface of the obturator internus and the origin of the levator ani muscle. Previously, we demonstrated that these two muscles are broadly connected via the obturator fascia and that this interface constitutes the origin of the levator ani (Muro et al., 2023), suggesting that the structural properties of the obturator internus may influence the morphological environment of the levator ani origin. In the present study, this groove progressively shifted downward with age. Such age‐related shape variation may alter the morphological configuration of the levator ani origin and warrants further investigation regarding its potential functional implications.

Our findings suggest that the structural integrity of the hip region, including the obturator internus, may be important for maintaining pelvic floor support. Tuttle et al. demonstrated that targeted strengthening of the obturator internus increased peak contraction pressure of the pelvic floor muscles, suggesting a functional contribution of the hip musculature to pelvic floor performance (Tuttle et al., 2016). Conversely, other interventions combining hip adduction and abduction exercises failed to alter vaginal pressure (Amorim et al., 2017), suggesting that not all hip movements effectively enhance pelvic floor function. Hwang et al. reported no significant correlation between hip muscle strength and pelvic floor muscle function in women with stress urinary incontinence (Hwang et al., 2021). These findings imply that although adequate hip function is a prerequisite, it does not suffice on its own. Given its anatomical location and fascial connectivity, the obturator internus likely plays a central mediating role in this functional linkage (Muro et al., 2023). Senkaya et al. reported a significant negative correlation between fetal weight and obturator internus cross‐sectional area (Senkaya et al., 2023), suggesting that the obturator internus may undergo structural remodeling in response to childbirth. These findings indicate that the functional status of the obturator internus is influenced not only by aging but also by reproductive history.

The findings of the present study advance the anatomical understanding of age‐associated pelvic floor muscle morphology. However, because clinical symptoms and dynamic function were not assessed, the present data do not establish diagnostic, preventive, or therapeutic implications. Future studies may benefit from integrating muscular morphology with functional measurements, ligamentous and connective tissue support, obstetric history, and clinical outcomes.

This study had several limitations. First, the sample was limited to adult women without clinically evident pelvic floor disorders, and obstetric history was not consistently available; therefore, the effects of vaginal childbirth could not be evaluated. Second, the evaluation was based solely on static MRI images, which do not capture the dynamic functional interplay between muscle contraction and movement. Third, landmark placement was performed by a single evaluator without formal inter‐ or intra‐observer reliability testing. Consequently, landmark‐placement accuracy and reproducibility cannot be formally quantified, and the findings require validation by independent evaluators and datasets. Fourth, ligamentous and other connective tissue structures were not evaluated, precluding causal inferences about their relationships with the observed muscular morphology or with pelvic floor symptoms. Fifth, because this was a cross‐sectional study, longitudinal age‐related change could not be observed directly. Finally, although participants were limited to women with benign gynecological diseases unrelated to pelvic floor dysfunction, these conditions may be associated with age or hormonal status and could potentially confound the observed age‐shape associations. Future studies including healthy volunteers and stratified analyses according to obstetric, hormonal, or disease status would help clarify these effects.

## 5 CONCLUSIONS

This study identified age‐associated morphological differences in the obturator internus and levator ani muscles. Inferiorly oriented shape variation of the groove and medial eminence on the medial surface of the obturator internus occurred in parallel with verticalization of the levator ani and widening of the levator hiatus. These coordinated associations provide a morphological framework for future studies integrating muscular shape, pelvic floor function, ligamentous and connective tissue support, and clinical outcomes.

## AUTHOR CONTRIBUTIONS

Conceptualization: Satoru Muro and Akimoto Nimura; Formal analysis and investigation: Satoru Muro, Junichi Tsuchiya, Takuya Ibara; Writing—original draft preparation: Satoru Muro; Writing—review and editing: Satoru Muro, Akimoto Nimura, Keiichi Akita; Supervision: Keiichi Akita. All authors read and approved the final manuscript.

## Data Availability

The data that support the findings of this study are available from the corresponding author upon reasonable request. Source code and documentation for Seg and Ref/SegRef3D are available at https://github.com/SatoruMuro/SegRef3D. The remaining analysis protocols and software are described in the Materials and Methods and Figure 1.
